# Resveratrol Modulates the Inflammatory Profile of Immune Responses and Circulating Endothelial Cells’ (CECs’) Population During Acute Whole Body Gamma Irradiation

**DOI:** 10.3389/fphar.2020.528400

**Published:** 2020-09-04

**Authors:** Ayman Khalil, Ghassan Al-Massarani, Abdulmunim Aljapawe, Adnan Ekhtiar, M. Adel Bakir

**Affiliations:** ^1^ Human Nutrition Laboratory, Department of Radiation Medicine, Atomic Energy Commission of Syria (AECS), Damascus, Syria; ^2^ Biomarkers Laboratory, Radiation Medicine Department, Atomic Energy Commission of Syria (AECS), Damascus, Syria; ^3^ Flow Cytometry Laboratory, Biotechnology and Molecular Biology Department, Atomic Energy Commission of Syria (AECS), Damascus, Syria; ^4^ Radiation Medicine Department, Atomic Commission of Syria (AECS), Damascus, Syria

**Keywords:** resveratrol, whole body gamma irradiation, Kupffer cells, IL10, hepatic cells, circulating endothelial cells****

## Abstract

Wistar rats were whole body irradiated with a single dose of 2 Gy post administration with 10 or 100 mg/kg of resveratrol (RSV) intraperitoneally for 30 days. Rats’ livers were dissected and processed to analyze immune response profiles of Th1, Th2, Th9, Th17, and Th22 by flow cytometry. In addition, peripheral blood samples were collected and circulating endothelial cells (CECs) were counted as an indicator for endothelial damage. Results demonstrated that resveratrol at 100 mg/kg enhanced liver immunological response influenced by irradiation by inducing Th2 immune response that was revealed by an increase in IL-10 secretion to more than 5,000 pmol/ml post irradiation. Results also indicated that RSV, at a dose of 100 mg/kg, decreased levels of the main pro-inflammatory cytokines such as INF-*γ*, IL-22, IL-17A, and GM-CSF post irradiation. In addition, the same RSV was bound to upregulate the expression of IL-10 mRNA in isolated Kupffer cells (KCs) and their secretion of IL-10 post irradiation. The result demonstrated that KCs were the central source of this anti-inflammatory response mediated mainly by IL10. These results, proposed for the first time, clearly states that RSV promotes IL-10 mediated immune resolution by Kupffer cells and not by hepatocytes. This implies that KCs have a crucial role in radiotherapy. Additionally, this study showed that RSV had an anti-apoptotic effect through re-increasing the number of CECs, which is implicated in irradiation damage. Result of the current work discloses novel findings about the potential of RSV as a radio-protector agent of a natural origin and suggests novel roles of KCs as a pharmacological target during radiation exposure.

## Introduction

Radiotherapy is an indispensable method for cancer therapy (Hogle, 2007). However, its adversarial effects may progress during the course of therapy. In order to protect against the harmful effects of irradiation exposure on normal tissues, many agents were investigated (Citrin et al., 2010). Attempts to protect the normal tissues from the deleterious effects of ionizing radiations through pharmacological interventions were attempted as early as 1949, and the search for new radio-protectors continues (Jagetia, 2007). In fact, numerous radio-protective compounds are the target of several studies aiming at exploring their role as radio-protectors during cancer radiotherapy (Jagetia et al., 2003; Maurya et al., 2007). Actually, recognizing the balance between therapy effectiveness and radiation’s adverse effects is the main purpose of radiotherapy (Xiaoxin et al., 1997). Thus, different synthetic compounds have been studied both *in vitro* and *in vivo*. However, the toxicological effects of these agents at the protective dose prevented their clinical utilization (Weiss and Landauer, 2000). Therefore, focus of radiation protection has shifted to test the radio-protective potential of natural compounds hoping to find suitable pharmaceuticals that could protect humans against the deleterious effects of ionizing radiation in clinical and radiation incidents (Citrin et al., 2010; Hosseinimehr et al., 2011). Resveratrol (RSV) (3, 4, 5-trihydroxystilbene) is a polyphenol stilbene compound that is found naturally in red wine and in skin of red fruits and vegetables such as cabbages, berries, and peanuts (Jang et al., 1997; Burns et al., 2002; Singh et al., 2007; Guan et al., 2012). It has anti-inflammatory, anti-oxidant, and anti-carcinogenic proprieties (Aggarwal et al., 2004; Donnelly et al., 2004; Kakoti et al., 2015). Resveratrol as a natural compound received innumerable considerations as a radio-protector (Sebastià et al., 2013; Sebastià et al., 2014; Ocolotobiche et al., 2019). In fact, RSV seems to inhibit the inflammatory responses post liver irradiation by suppressing the increase in plasma tumor necrosis factor-α (TNF-α) concentrations (Aggarwal et al., 2004). In addition, RSV protects blood lymphocytes against radiation-induced damage (2 Gy of gamma irradiation) in mice (Koohian et al., 2017) and reduces free radical levels resulting from ionizing radiation injuries, which provides a type of protection by promoting the activities of antioxidant enzymes such as superoxide dismutase and catalase (Losa, 2003; Signorelli and Ghidoni, 2005). Moreover, RSV protects the hepatic irradiation-induced damage through its anti-oxidant activity and its capacity to reverse histopathological alterations induced by irradiation (Velioglu-Ogunc et al., 2009). In fact, RSV can play an essential role in the prevention and treatment of liver disorders by reducing hepatic fibrosis (Faghihzadeh et al., 2015).

Carsten et al. (2008) reported that the radio-protective effects of RSV at a daily dose of 100 mg/kg administrated orally 2 days prior to 3 Gy whole body gamma irradiation and continued up to 30 days, reduced chromosome aberrations significantly in bone marrow of irradiated mice. Correspondingly, the daily dose of RSV for 30 days did not show any chromosomal aberrations in non-irradiated mice. RSV also inhibits inflammation induced by ionizing radiation in mesenchymal stem cells (MSCs) through activating Sirt1 pathway and downregulating of IL-1*β* derived from NLRP-3 inflammatory activated after MSC irradiation (Fu et al., 2013). RSV administration for 21 days post irradiation resulted in an increased Sirt1 mRNA expression (Li et al., 2014). In fact, several studies have shown that RSV can act as a potent radio-protector (Thekkekkara et al., 2017). RSV enhances the radio sensitivity of cancer cell lines to ionizing radiation, suggesting a possible clinical benefit for patients receiving radiotherapy (Zoberi et al., 2002; Baatout et al., 2004; Fiore et al., 2005; Scarlatti et al., 2007; Johnson et al., 2008).

RSV also controls immunity response by interfering with immune response regulation, pro-inflammatory cytokine synthesis, and gene expression in immune cells. RSV targets several inflammatory components and exerts immune-regulatory effects on immune cells (Multhoff and Radons, 2012; Malaguarnera, 2019). Numerous important mediators in the cell are targets of RSV such as sirtuins, adenosine monophosphate kinase (AMP), NF-*κ*B, inflammatory cytokines, and cellular processes such as gluconeogenesis, lipid metabolism, mitochondrial biogenesis, angiogenesis, and apoptosis (Malaguarnera, 2019).

Despite the numerous studies demonstrating the possible role of RSV as a radio-protective compound, little is known about its ability to modify the effect of radiation in normal and cancer cells (Dobrzynska, 2013). Thus, in the current study, the ability of resveratrol to modulate the effect of radiation in normal tissue post whole body gamma irradiation was thoroughly investigated. RSV effects on the number of circulating endothelial cells (CECs), which is increased during irradiation therapy and considered as a marker of apoptosis, was also investigated in this work.

## Material and Methods

### Animals

Thirty-six male Wistar rats, weighing 100 g, aged 8 weeks, were included in this study and handled according to the guidelines of the Local Scientific and Ethical Committee of the Atomic Energy Commission of Syria (AECS), Damascus, Syria (permit document 2-28/10/2018), which is derivative of the European Community guidelines for the care and management of laboratory animals. Rats were divided into six groups, six rats each, as follows: (1) control group (C); (2) rats irradiated with a dose of 2 Gy with no RSV administration (C/IR); (3) rats administered with 10 mg/kg of RSV (RSV10); (4) rats administered with 100 mg/kg RSV (RSV100); (5) rats administered with 10 mg/kg RSV followed by 2 Gy irradiation (2G/RSV10); (6) rats administered with 100 mg/kg RSV followed by 2 Gy irradiation (2G/RSV100). RSV was administered intraperitonealy (I.P.) every 24 h for 30 days prior to irradiation. Rats were fed and kept under optimal conditions (50–60% relative humidity and 22°C temperature) in stainless steel cages till the end of the study.

### Animal Irradiation Procedure

Animals were exposed to whole-body gamma irradiation at a dose rate of 100 mGy/m using a gamma ray apparatus (Theratron 80 Canadian design machine, ^60^Co, focal distance of 100 cm) (Khalil and Al-Daoude, 2019). Irradiation protocol was approved by the Atomic Energy Commission of Syria according to the IAEA regulations and safety measures.

72 h post-irradiation, rats were deprived of food overnight and then anesthetized with ether. The abdomen was opened through a midline incision; then a 25G needle was inserted in the inferior vena cava to collect blood into a citrate containing tube for the immuno-magnetic separation (IMS) analysis of CECs; then, the animals were sacrificed *via* cervical dislocation and the liver was dissected, rinsed with PBS. Liver specimen of each rat was cut into five parts; one part was embedded in cryostat embedding medium for histological study, and the rest were stored at −80°C for other assays.

### Cytokine Quantification by Flow Cytometry

Hepatocytes and Kupffer cells isolated from each liver sample were rinsed with PBS, then suspended in 1 ml of Tris-Urea-CHAPS lysing buffer (50 mM Tris.Cl, pH 7.4, 8.75 M urea, 2.5 M thiourea, 5% (w/v) CHAPS, 50 mM dithiothreitol, and 0.1 mM phenymethanesulfonyl fluoride (PMSF) which was added immediately before use). The tissue lysate was clarified by centrifugation for 30 min at 14,000× g/4°C. Supernatant was directly used to quantify the cytokines using Rat Th Cytokine panel (13-plex) kit, (BioLegend^®^, Cat. No. 740283). The kit is designed to quantify 13 cytokines; namely: IL-10, IFN-*γ*, IL-5, IL-2, TNF-*α*, GM-CSF, IL-4, IL-17F, IL-9, IL-17A, IL-13, IL-22, and IL-6 and was used according to the manufacturer’s instructions and guidelines. Briefly, 25 µl of the cleared supernatant sample was incubated with beads coated with each anti-cytokine antibodies then washed and incubated with detection antibodies before washing and analyzing. BD Biosciences FACSCalibur flow cytometer was set and calibrated to acquire data according to the manufacturer’s instructions. Standard cytokine panel cocktail was utilized to build the standard curves, which were then used to calculate cytokine concentrations. BD Biosciences FCAP software was deployed to calculate cytokine concentrations. Three replicates of each liver tissue sample were measured.

### Hepatocytes and Kupffer Cell Isolation

Hepatocytes and Kupffer cells were isolated from Wistar rat livers according to a modified method previously described by Shulman and Nahmias (2013). Frozen liver tissue samples were brought into room temperature and rinsed with PBS, then each liver sample (weighing 50 mg) was minced in a sterile petri dish containing 5 ml of collagenase dissolved in Krebs Ringer Buffer (0.105 g collagenase type IV from *clostridium histolyticum* (Sigma-Aldrich^®^) and 2 g of CaCl_2_·H_2_O were added to 150 ml of Krebs Ringer Buffer). Krebs Ringer Buffer recipe: 28.54 g NaCl, 3.98 g D-Glucose, 8.40 g NaHCO_3_, 1.68 g KCl, and 19.06 g HEPES dissolved in 4 L ultrapure water, pH 7.2–7.4, filtered through 0.22 µm filter, and stored at 4°C. Cell suspension was passed through a 70 µm pore mesh filter into 50 ml conical tubes and centrifuged at 40 × g/4°C for 5 min. Then cell pellet was resuspended in 25 ml of salt-balanced Percoll solution, mixed gently, and centrifuged at 500× g/4°C for 10 min. Kupffer cells recovered from the band in the supernatant, and hepatocytes recovered from the pellet. Cells were then washed with PBS and used in other assays.

### Total mRNA Extraction and Real Time PCR

Total RNA was extracted from isolated hepatocytes and Kupffer cells of rat liver samples using Roti-phenol reagent (Phenol/Chloroform/Isomylalcohol: 25/24/1, ROTH^®^, Germany), and quantified by NanoDrop (NanoDrop technologies, Qiagen, Germany). The total RNA (1 µg) from each cell type was amplified using the 2× PCR Sy Greenone-step low Rox kit according to the manufacturer’s recommendations (ROTH^®^, Germany). PCR was performed using 0.5 µM of each primer and with cycling conditions as follows: 10 min denaturing at 95°C, followed by 40 cycles of 30 s denaturing at 95°C, 30 s primers annealing at 60°C and 30 s fragment elongation at 72°C.

Mx3OO5P QPCR system (Agilent technologies, Germany) was used to quantify the expression of target genes according to the procedure described in previous works (Khalil et al., 2018; Khalil and Al-Daoude, 2019). Data were quantified by the method of 2^−^ΔΔCt. Primer sequences (Eurofins Genomics, Germany) for the reference and target genes were:

*β*-Actin: 5′-AAGGCCAACCGTGAAAAGAT-3′; 5′-TGGTACGACCAGAGGCATAC-3′

IL-10: 5′-CACAAAGCAGCCTTGCAGAA-3′; 5′-AGAGCAGGCAGGATAGCAGTG-3′

### Measurement of IL10 Levels of Hepatocytes and Kupffer Cells

Total protein was extracted from isolated hepatocytes and Kupffer cells by adding 1 ml of ice-cold nondenaturing lysis buffer to 0.5–1 × 10^7^ cells of each cell type in a separate 15-ml capped conical tubes. The nondenaturing lysis buffer receipe is 1% Triton X-100, 50 mM Tris.Cl, pH 7.4, 300 mM NaCl, 5 mM EDTA, 0.02% sodium azide, 10 mM iodoacetamide, and 1 mM PMSF). Iodoacetamide, PMSF, and leupeptin (2 μg/ml) were added immediately before use. Cells were suspended in lysis buffer by gentle agitation for seconds with a vortex mixer set at medium speed, then the suspension was kept on ice for 15 to 30 min. Cell lysate was then cleared by centrifugation for 15 min at 14,000× g/4°C, and the supernatant was used to measure IL-10 levels. IL-10 levels were measured using Interleukin-10 ELISA kit (Sigma Aldrich^®^, St. Louis, MO 63103 USA) according to the manufacturer’s instructions as described in a previous work (Khalil et al., 2018).

### Histological Study of the Liver

Liver specimen embedded in cryostat embedding medium (Killik, Bio-Optica, Italy) and frozen at −80°C was processed as described in previous work (Khalil et al., 2018). Specimens were cut into 5 µm thick sections by cryostat (SLEE MNT Cryostat Medical GmbH, Germany), then stained with hematoxylin (Flukachemie Gm bHcH, Switzerland) and eosine (Qualikems laboratory Reagent, India) according to standard histological staining procedures. Stained liver tissues were imaged using a light microscope (BX53, Olympus, Japan) connected to a personal computer.

### Detection and Quantification of Circulating Endothelial Cells

Endothelial cells were isolated from the whole blood using Dynal M450 IgG1 immune magnetic beads (Dynal AS, Oslo, Norway). 4.5 µm diameter beads were coated with affinity-purified pan-anti-mouse immunoglobulin G1; 10 µl of rabbit monoclonal antibody against rat CD146 (10 µg/Ml EPR3208, Abcam, Cambridge, UK) was diluted to 100 µl with PBS [(containing 0.1% bovine serum albumin (BSA) and 0.1% sodium azide]. 100 µl of magnetic bead suspension was incubated overnight with the diluted anti-CD146 antibody solution at 4°C with head-over-head agitation. Beads were then washed three times with PBS–BSA–NaN3 to remove excess of antibodies then resuspended with buffer until use. 1 ml of the blood was diluted 1:3 with the isolation buffer and incubated for 1.5 h at 4°C on a rotator with 15 µl of the prepared magnetic beads.

Separation of beads rosetted with endothelial cells from the blood samples required a minimum of 2 min exposure to the magnet. Three washes were performed to completely remove non-rosetted cells. After the third wash, the rosetted cells were recovered in a 100 µl solution of acridine orange (at a final concentration of 5 µg/ml in PBS). Endothelial cells were counted using 0.5 mm Nageotte chamber cytometer (Hecht-Assistent, Sondheim, Germany) and fluorescent microscope (BX51, Olympus, Japan) equipped with a 500/20 nm excitation filter. The quality of images was improved by Deltapix software version 1.6 (Deltapix, Måløv, Denmark).

### Statistical Analysis

All experiments were repeated three times, and data were statistically analyzed using a Mann–Whitney U test and presented as the mean ± SD. All statistical analyses were performed with GraphPad Prism 7.0 (GraphPad Software, San Diego, USA), and a p value of 0.05 was considered of statistical significance. For endothelial cell quantitation, the results were presented as Mean cell number ± SEM.

## Results

### Effect of Resveratrol on Th1 Immune Response (IL-2, TNF-α, and INF-*γ*) in Non-Irradiated Rats and in Rats After Acute Whole Body Irradiation

**Figure 1:**
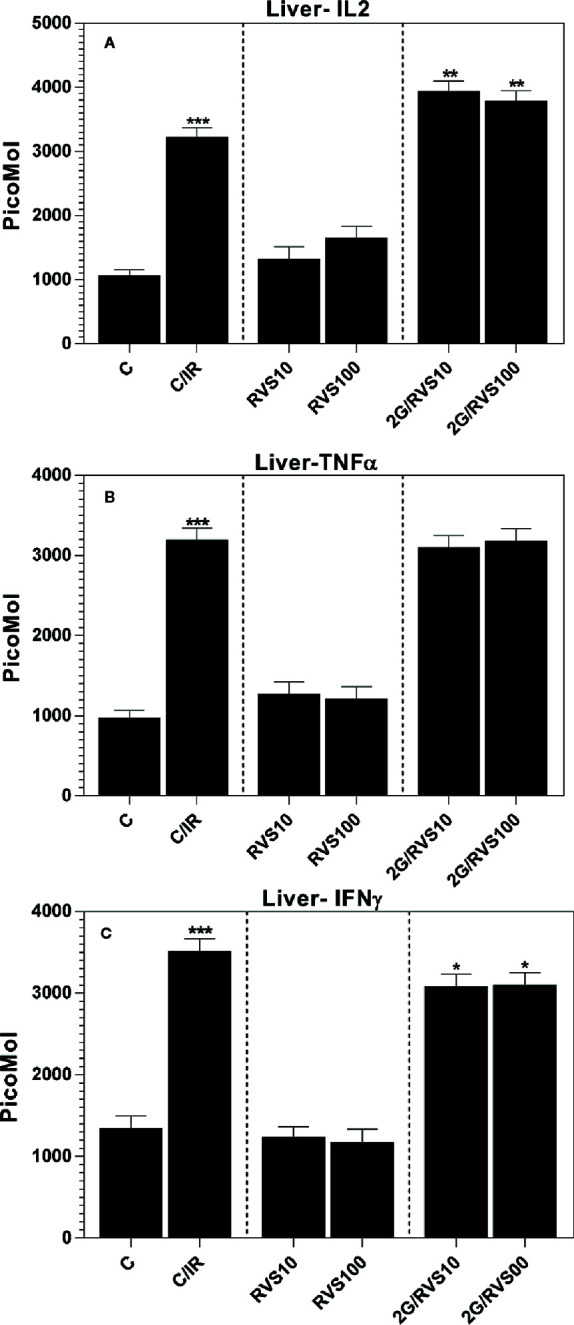
The cytokine profiles of Th1 cells measured in total protein extract of Wistar rat liver: **(A)** IL-2, **(B)** TNF-α, and **(C)** INF-*γ* in six rat groups (n = 3). (C, control group; C/IR, irradiated rats, RSV10 and RSV100; irradiated rats with 2 Gy pretreated with resveratrol 10 mg and 100 mg/kg body weight, respectively). C/IR are compared to C; RSV10 or RSV100 are compared to C; 2G/RSV10 or 2G/RSV100 are compared to C/IR. *p < 0.05, **p < 0.01, ***p < 0.001.

Resveratrol at doses of 10 or 100 mg/kg had no effect on the Th1 immune response (IL-2, TNF-α, and INF-*γ* levels) in non-irradiated rats (**Figures 1A–C**) compared to non-irradiated non-resveratrol treated control group (C). Irradiated rats (C/IR group) showed a significant increase in all measured Th1 cytokines (IL-2, TNF-α, and INF-*γ*) by about 3-, 3.3- and 2.6-fold, (p ≤ 0.05), respectively, compared to non-irradiated non-resveratrol treated control (C group), (**Figures 1A–C**). In irradiated rats administrated with 10 or 100 mg/kg RSV, IL-2 level increased significantly by 1.2-fold (p ≤ 0.05), (**Figure 1A**) compared to irradiated non-resveratrol treated control (C/IR). In contrast, RSV at 10 or 100 mg/kg decreased INF-*γ* levels by about 0.9-fold (p ≤ 0.05) compared to the C/IR group (**Figure 1C**). No significant alterations in TNF-α levels were observed due to RSV administration in irradiated rats compared to the C/IR group (**Figure 1B**).

### Effect of Resveratrol on Th2 Immune Response (IL4, IL5, IL6, IL10, and IL13) in Non-Irradiated Rats and in Irradiated Rats

**Figure 2:**
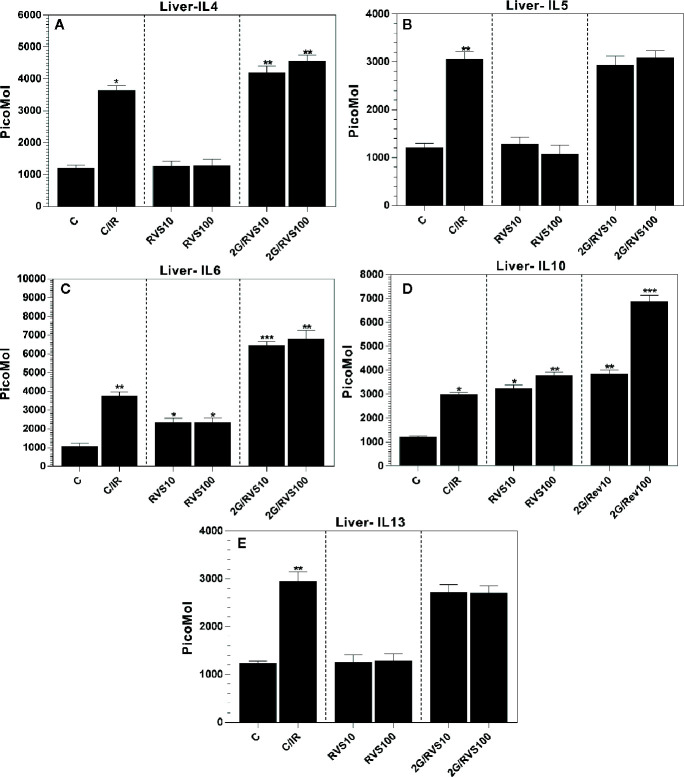
The cytokine profiles in Wistar rat liver produced by Th2 cells, which include **(A)** IL-4, **(B)** IL-5, **(C)** IL-6, **(D)** IL10, and **(E)** IL-13 in six rat groups. (C, control group; C/IR, irradiated rats, RSV10 and RSV100; irradiated rats with 2 Gy pretreated with resveratrol 10 mg 100 mg/kg body weight, respectively). C/IR are compared to C; RSV10 or RSV100 are compared to C; 2G/RSV10 or 2G/RSV100 are compared to C/IR. *p < 0.05, **p < 0.01, ***p < 0.001.

RSV at doses of 10 or 100 mg/kg increased IL-6 levels by 2.2-fold (p ≤ 0.05) compared to non-irradiated non-resveratrol treated control (C group) (**Figure 2C**). In addition, RSV administration increased IL-10 levels significantly by approximately 2.7- and 3.2-fold (p ≤ 0.05) at 10 mg/kg (RSV10) and 100 mg/kg (RSV100) compared to a non-irradiated non-resveratrol treated control (C group) (**Figure 2D**). No alterations in IL-4, IL-5, and IL-13 levels were observed after administrating RSV at any used dose (**Figures 2A, B, E**). A single gamma irradiation dose of 2 Gy (C/IR group) indicated an increase in all measured Th2 immune response cytokines as shown in **Figure 2**. However, our results showed that IL-4, IL-5, IL-6, IL-10, and IL-13 levels were significantly increased in irradiated rats compared to non-irradiated non-resveratrol treated control (C group). This increase was ~3-fold (**Figure 2A**), 2.5-fold (**Figure 2B**), and 3.5-fold (**Figure 2C**), 2.6-fold (**Figure 2D**) and 2.5-fold (p ≤ 0.05) for IL-4, IL-5, IL-6, IL-10, and IL-13 (**Figure 2E**), respectively. In addition, administration of 10 mg/kg or 100 mg/kg of RSV before irradiation increased the expression of IL-4 by 1.6-, 1.7-fold (p ≤ 0.05) and IL6 by 1.7-, 1.8-fold (p ≤ 0.05) (**Figures 2A, C**), respectively compared to irradiated non-RSV treated rats (C/IR group). Similarly, administration of 10 mg/kg or 100 mg/kg of RSV before irradiation significantly increased the expression of IL-10 levels by about 1.3- and 2.31-fold (p ≤ 0.05) respectively, as demonstrated in **Figure 2D**. In contrast, RSV at 10 mg/kg or 100 mg/kg dose did not alter the expression of IL-5 or IL-13 (**Figures 2B, E**).

### Effect of Resveratrol on Cytokine Profiles of Th9 (IL9), Th17 (IL17A, IL17F, and GM-CSF), and Th22 (IL22) in Non-Irradiated Rats and in Irradiated Rats

**Figure 3:**
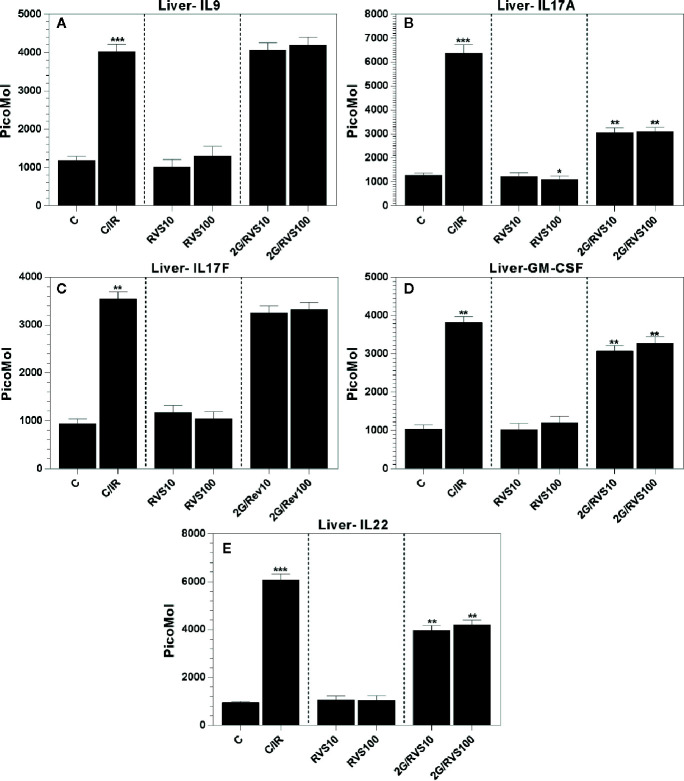
The cytokine profiles in Wistar rat liver produced by Th9,Th17, and Th22 cells which include **(A)** IL-9, **(B)** IL-17A, **(C)** IL-17F, **(D)** GM-CSF and **(E)** IL-22 in six rat groups. (C, control group; C/IR, irradiated rats, RSV10 and RSV100; irradiated rats with 2 Gy pretreated with resveratrol 10 mg 100 mg/kg body weight, respectively). C/IR are compared to C; RSV10 or RSV100 are compared to C; 2G/RSV10 or 2G/RSV100 are compared to C/IR. *p < 0.05, **p < 0.01, ***p < 0.001.

In comparison with non-irradiated non-resveratrol treated control (C group), RSV at 10 mg/kg or 100 mg/kg did not alter Th9, Th17, and Th22 cytokine profiles (**Figures 3A, C–E**) except for IL-17A which was reduced by 0.8-fold (p ≤ 0.05). However, gamma irradiation increased the levels of all measured cytokines in irradiated rats (C/IR group) compared to non-irradiated non-resveratrol treated control (C group). Whereas IL-9, IL-17 (A and F), GM-CSF, and IL-22 significantly increased by about 3.3-, 4.8-, 3.7-, 3.5-, and 6.3-fold, (p ≤ 0.05), respectively (**Figure 3**). Contrarily, the treatment with 10 mg/kg or 100 mg/kg RSV prior to irradiation did not alter IL-9 and IL-17F expressions (**Figures 3A, C**). IL-17A and GM-CSF which enhance pro-inflammatory cytokine production (Brembilla et al., 2018; Hamilton, 2019) were downregulated by about 0.5- and 0.8-fold (p ≤ 0.05), respectively, in irradiated rats pretreated with 10 mg/kg or 100 mg/kg RSV (**Figures 3A, D**) compared to non-RSV treated irradiated rats (C/IR). The same result was obtained for IL-22, which somehow decreased by about 0.7-fold (p ≤ 0.05) in irradiated rats treated with both RSV concentrations (**Figure 3E**).

### Upregulation of IL-10 mRNA Expression and Increase of IL-10 Concentrations in Isolated Rats Hepatocytes and Kupffer Cells After Resveratrol Administration

**Figure 4:**
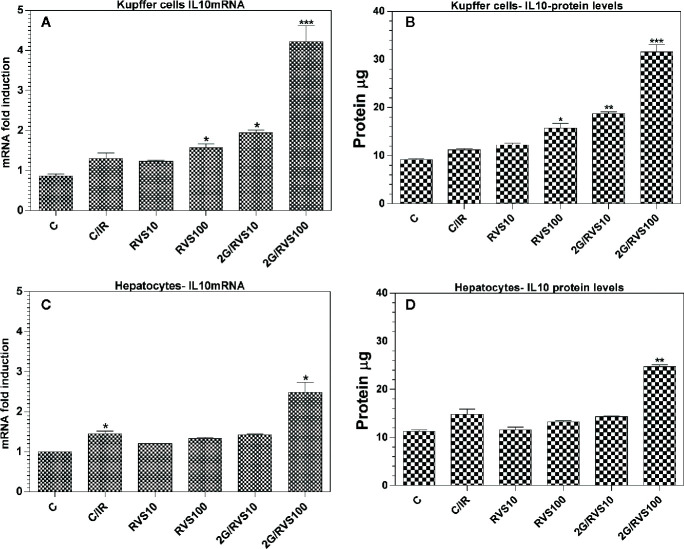
Effects of a 2 Gy single dose of whole body irradiation and RSV on IL10 relative mRNA gene expression and protein levels in Hepatocytes **(A**, **C)** and Kuppfer cells **(B, D)** of Wistar rats. (C, control group; C/IR, irradiated rats, RSV10 and RSV100; irradiated rats with 2 Gy pretreated with resveratrol 10 mg 100 mg/kg body weight, respectively). C/IR are compared to C; RSV10 or RSV100 are compared to C; 2G/RSV10 or 2G/RSV100 are compared to C/IR. *p < 0.05, **p < 0.01, ***p < 0.001.

IL-10 is a very important anti-inflammatory cytokine, which maintains normal tissue homeostasis (Iyer and Cheng, 2012). The results of the current study found that RSV induces the production of IL-10, the most significant anti-inflammatory cytokine among all measured cytokines. In order to identify the origin of this important anti-inflammatory cytokine induced by RSV administration, we isolated the hepatocytes and Kupffer cells. RSV increased IL-10 mRNA expression and IL-10 levels in both hepatocytes and Kupffer cells. As validated in **Figures 4A, C**, the IL-10 mRNA expression in both Kupffer (KCs) and hepatocytes (HCs) cells isolated from livers of irradiated rats pretreated with 100 mg/kg RSV increased by about 3.3-and 2.5-fold (p ≤ 0.05), respectively (**Figures 4A, C**). This increase is further confirmed by an increase in IL10 protein levels extracted from isolated cells (**Figure 4B**). RSV also triggered a significant increase in IL-10 protein level in HCs, solely in rats pretreated with 100 mg/kg of RSV (**Figure 4D**). **Figures 4B, D** clearly show that in irradiated rats pretreated with 100 mg/kg of RSV, IL-10 levels produced by KCs increased significantly compared to IL-10 levels generated by KCs isolated from livers of irradiated rats non-treated with RSV (C/IR group). The concentrations of IL-10 in KCs and in HCs isolated from livers of irradiated rats pretreated with RSV increased by about 2.6- and 1.6-fold (p ≤ 0.05), respectively compared to irradiated rats (C/IR group). This implies that production of IL-10 by KCs in irradiated rats pretreated with 100 mg/kg of RSV was about twofold higher.

### Resveratrol Up-Regulates the Number of Kupffer Cells Post-Irradiation

**Figure 5:**
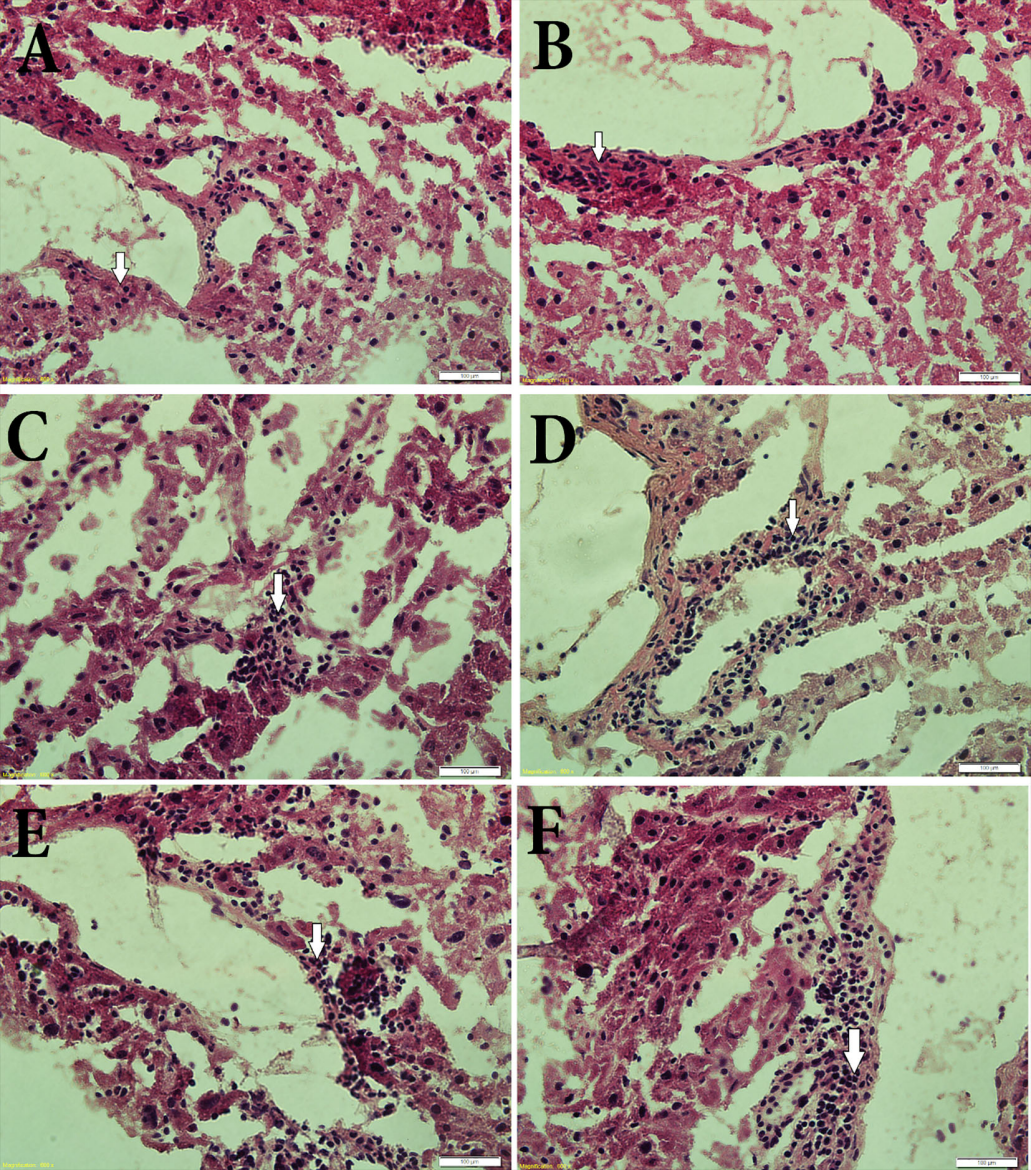
Histological sections of parts of rat liver specimens: **(A)** control (non-irradiated non-treated with RSV), **(B)** 2 Gy irradiated control and non-treated with RSV, **(C)** non-irradiated treated with 10 mg/kg body weight RSV. **(D)** non-irradiated treated with 100 mg/kg body weight RSV. **(E)** 2 Gy irradiated pretreated with 10 mg/kg body weight RSV. **(F)** 2 Gy irradiated, pretreated with 100 mg/kg body weight RSV. Sections show an obvious increase in Kupffer cell boundaries of blood vessels due to pretreatment with 100 mg/kg body weight RSV.

A histological study on liver tissues was conducted to evaluate the effects of RSV on the histology of hepatic tissue and on the number of Kupffer cells, which are the most abundant immune cells in the liver. **Figure 5** demonstrates that irradiation increased the number of Kupffer cells (**Figure 5B**) in the liver of irradiated rats compared to the non-irradiated control (**Figure 5A**). It also demonstrates that Kupffer cell infiltration around the blood vessels increases after administrating rats with RSV at 10 mg/kg or 100 mg/kg in a dose dependent manner in non-irradiated and irradiated rats (**Figures 5C, D**).

### Resveratrol Pretreatment Enhanced the Number of Circulating Endothelial Cells (CECs) in Irradiated Rats

**Figure 6:**
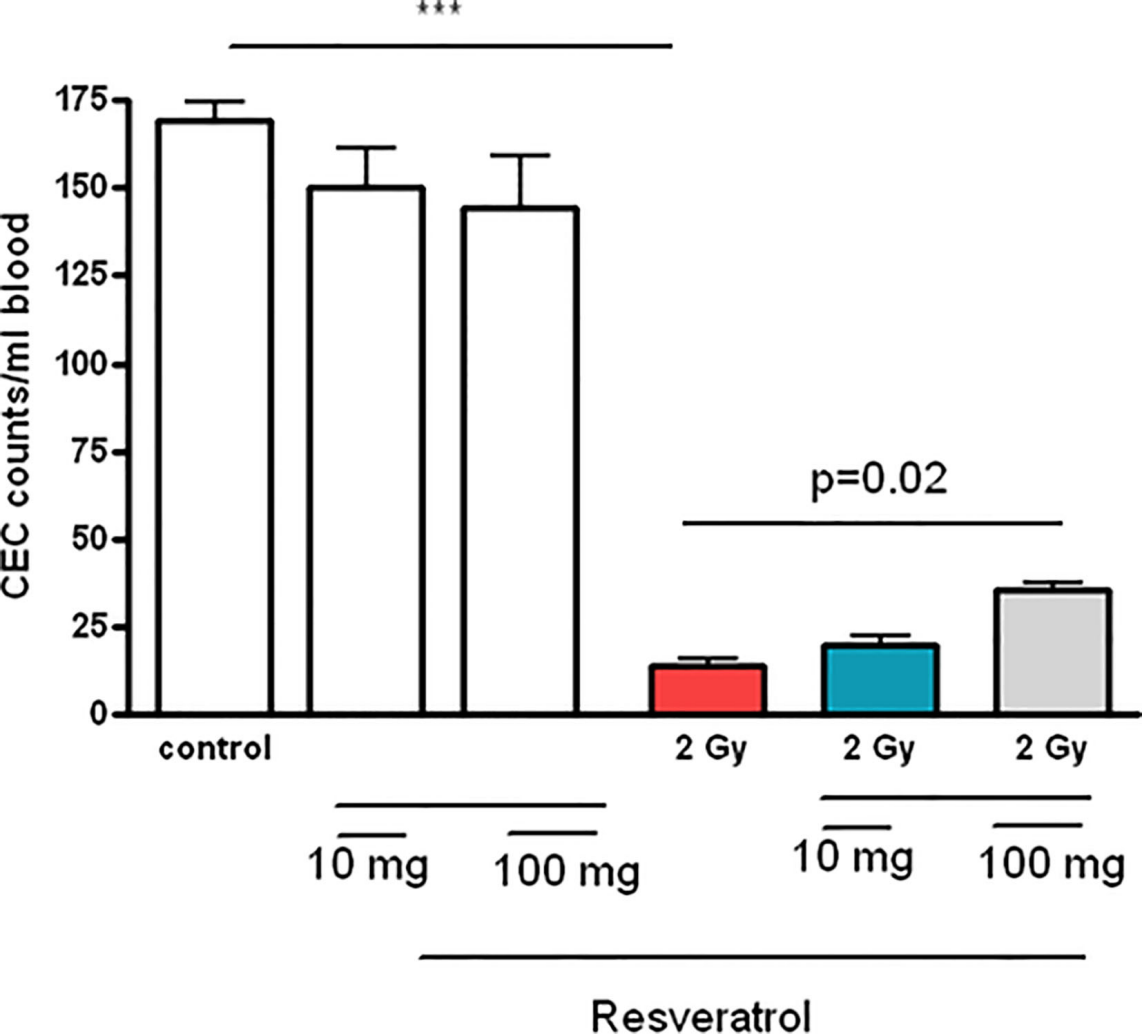
Effects of resveratrol treatment (10 and 100 mg/kg body weight) on the CEC counts of peripheral blood after rats’ irradiation with 2 Gy ***p < 0.001. Pretreatment with resveratrol enhanced the CEC counts in peripheral blood, which implies radioprotective effect.

A whole body irradiation of rats with a single dose of 2 Gy decreased the number of circulating endothelial cells (CECs) significantly after one day of irradiation compared to non-irradiated control group (mean ± SEM: 17 ± 3 cells/ml *vs* 165 ± 39 cells/ml, p ≤ 0.05). Resveratrol pretreatment enhanced the number of CECs in irradiated rats and increased significantly in rats pretreated with RSV and irradiated compared to irradiated group non-treated with RSV (mean ± SEM: 50 ± 5 cells/ml *vs* 17 ± 3 cells/ml, p ≤ 0.05). However, this increase has not reached CECs’ normal levels in non-irradiated rats (**Figure 6**).

## Discussion

Radiation exposure has been reported to induce several cytokines and growth factors *in vivo* and *in vitro* such as TNF-α, IL-1*β*, IFN-*γ*, GM-CSF, IL-4, IL-5, IL-6, IL-10, IL-12, and IL-18, and TGF-*β* (Zhang et al., 1994; Chiang et al., 1997; Hong et al., 1999; Beetz et al., 2000; Daigle et al., 2001; Ogawa et al., 2002; Han et al., 2006; Burnette et al., 2011; Schaue et al., 2012; Milgrom et al., 2016). IL-2, TNF-α, and INF-*γ* are pro-inflammatory cytokines produced by Th1 cells and amplifying the inflammatory response (Martin et al., 1997; Shan et al., 2007). These cytokines were upregulated by irradiation with 2 Gy dose of gamma radiation, and this result was expected as irradiation leads to upregulation of pro-inflammatory cytokines such as TNF-α, IL-1*β*, INF-*γ*, IL-6 (Shan et al., 2007). RSV administration before irradiation resulted in a slight inhibition of TNF-α production (**Figure 1B**). It also reduced the levels of INF-*γ* significantly compared to irradiated non-resveratrol treated control (**Figure 1C**). This is consistent with a previous study in which resveratrol could significantly reduce the levels of pro-inflammatory factors: TNF-α, IL-1*β*, and IL-*6* and greatly promote the expression level of SIRT1 pathway, which is the main pathway of pro-inflammatory inhibition by RSV (Schaue et al., 2012). However, in our experiments, IL-2 generated by Th1 cells and IL-6 produced mainly by Th2 cells were the only pro-inflammatory which was upregulated by RSV in rat liver subjected to irradiation (**Figures 1A** and **2C**). In fact, this increase in IL-6 by administration of 100 mg/kg RSV was also observed in peripheral blood mononuclear cells isolated from patients with osteoarthritis and was dose-dependent (Tao et al., 2015). Also, it was demonstrated that mRNA and serum levels of IL-2, IL-6, and IL-10 increased in patients post oral administration of resveratrol (Multhoff and Radons, 2012). In addition, RSV was found to enhance the expression of IL-1*β* and IL-6 in the peripheral blood lymphocytes (Wendling et al., 2013). These results are in accordance with our results. In fact, the enhanced production of IL-1*β* and IL-6 characterizes a pro-inflammatory status contributing to T helper lymphocyte differentiation and function (BaGen et al., 2018), but it is also involved in tissue regeneration (Schwager et al., 2017). Thus, this increase in IL-6 and IL-2 may be involved in tissue repair and regeneration post-irradiation. Furthermore, the immune cells exposed to RSV in the vascular compartment expressing significant levels of IL-1*β* or IL-6 are triggered for the adaptive immune response (Malaguarnera, 2019). Thus, we thought that the increase of IL-6 in our study was beneficial because IL-6 promotes specific differentiation of naïve CD4+ T cells, consequently performing an important function in linking innate and acquired immune response (Malaguarnera, 2019); also, RSV affects the immune cells only for a limited time because of its short half-life in the blood (Mauer et al., 2014).

GM-CSF is a pro-inflammatory cytokine that acts at the interface between innate and adaptive immunity (Rutz et al., 2013). RSV also ominously reduces the production of this pro-inflammatory cytokine (**Figure 3D**) which is in agreement with the results reporting that RSV reduces the expression of GM-CSF in TNF-α activated human umbilical vein endothelial cells (Wendling et al., 2013).

RSV inhibits the production of IL-17 induced by irradiation in C/IR rats (**Figure 3B**). In fact, the Th17 cells play very important roles in autoimmune diseases, which are key initiators of pro-inflammatory responses through recruiting neutrophils and macrophages to injured tissues and *via* their production of IL-17, which in turn plays an important role in host defense against infection of extra cellular pathogens. Moreover, Th17 cells produce IL-22, which, similar to IL-17, is beneficial to the host in many inflammatory disorders (Schwager et al., 2017). However, depending on the target tissue, IL-22 can be pathogenic due to its inherent pro-inflammatory properties, which are further enhanced when IL-22 is released together with other pro-inflammatory cytokines, in particular IL-17 (Astry et al., 2015). In fact, RSV, in our study prevents the link of IL-17 and IL-22. Thus, RSV plays a protective role against the unexpected consequences from IL-22 and IL-17 operating together (**Figures 2B, E** **)**.

RSV exhibits an anti-inflammatory profile in macrophages (Walle et al., 2004; Murray et al., 2014; Astry et al., 2015), and the KCs are resident macrophages of the liver. Our results support this role of RSV in KCs of the liver by producing IL-10, which has a strong anti-inflammatory role in the immune response. RSV enhances hepatocytes as reported in the current study to produce IL-10 response, but as demonstrated in **Figures 2D** and **3**, the IL-10 production by HCs is less important *versus* production of IL-10 by KCs that might be due to the increased number of KCs by RSV effect (**Figure 5D**) exhorting KCs to produce more anti-inflammatory response mediated by IL-10 in our study (**Figures 4A, B**). However, this finding indicates that RSV may promote IL-10 mediated immune resolution as reported previously (Chung, 2001; Bonizzi and Karin, 2004; Ogawa et al., 2008; Murray et al., 2014; Wang et al., 2016; Alharris et al., 2018). Actually, this result is of a great importance and leads us to conclude that RSV effects after whole body irradiation are mainly *via* KCs derived-IL-10 and not *via* HCs. Moreover, IL-10 plays a very important role in mediating host anti-inflammatory response. Therefore, identifying the cellular sources of IL-10 in addition to the molecular mechanisms that regulate IL-10 expression is critical to developing therapeutic strategies directed against pathology associated with impaired IL-10 production (Iyer and Cheng, 2012). In fact, these results give a great essential key role to liver KCs in immune response post whole body irradiation as a cellular and molecular target of future pharmacological molecule such as RSV. Moreover, RSV enhances the expression of the anti-inflammatory cytokine IL-4 produced by Th2 cells (**Figure 2A**) post irradiation. In fact, this effect of RSV to enhance the anti-inflammatory response by IL-4 and IL-10 is in accordance with a similar study in mouse bone marrow mesenchymal stem cells for therapeutic purposes reported by other groups (Wang et al., 2016).

Regarding IL-5 and IL-13 produced by Th2 cells, our data presented in **Figures 2A, B, E** demonstrated that these cytokines were not affected by RSV administration; in contrast, RSV triggered an insignificant decrease of these cytokines. In fact, these results are in agreement with another study where RSV (100 mg/kg body weight) was found to reduce IL-5 and IL-13 expressions in mice with OVA-induced asthma (Murray et al., 2014).

Actually, our results agree with previous works on the effect of resveratrol in normal tissues (RSV10 and RSV 100) compared to controls (C). In fact, it seems that RSV promotes the production of IL-10 (**Figure 2D**) and decreases IL-17A-mediated induction of IL-6 (Hwang et al., 2004). This action of RSV against IL-6 production (**Figure 2C**), especially at a dose of 100 mg/kg, gives an important strong anti-inflammatory and immune role in maintaining body hemostasis and preventing chronic diseases through inhibition of STAT3 and NF-kB activation resulting from promoting IL-17 and IL-6 together (Hou et al., 2014; Oike et al., 2018).

In a previous study, our group reported a gradual decline in CEC count post irradiation (AL-Massarani and Almohamad, 2014). Originally, the results of this study (**Figure 6**) suggest that injection of RSV attenuates radiation-induced CEC apoptosis in rats. This is consistent with previous studies, which demonstrated theprotective role of RSV against apoptosis induced by radiation (Zhang et al., 2017). Thus, RSV exerts a radio-protective effect on endothelial cells. The dose of resveratrol used in our study is safely achievable in humans because the dose of 100 mg/kg/day resveratrol in rat is equivalent to 2 mg/kg/day in humans (Yang et al., 2008).

Taken together, these findings suggest that RSV particularly of 100 mg/kg dose is a radio-protective pharmacological compound, and liver KCs might be the cellular therapeutic targets of RSV during acute whole body irradiation. Furthermore, our findings clearly demonstrate that RSV can modulate CEC population, which is considered as a good marker of radiation exposure. RSV was found to partially re-populate these cells. CEC results support the use of RSV as a radio-protector with the potential for radiotherapy application.

## Data Availability Statement

The raw data supporting the conclusions of this article will be made available by the authors, without undue reservation, to any qualified researcher.

## Ethics Statement

All institutional and national guidelines for the care and use of laboratory animals were obeyed by the Local Scientific and Ethical Committee of the Atomic Energy Commission of Syria (AECS), Damascus, Syria (permit number is 2-28/10/2018). This article does not contain any studies with human participants performed by any of the authors.

## Author Contributions

AK supervised the study, analyzed all results and wrote the manuscript. GA-M designed the study and planned the part related to counting of CECs. MB was the scientific adviser and edited the final manuscript. AE and AA carried out the flow cytometry measurement and analyzed the related results. All authors contributed to the article and approved the submitted version.

## Conflict of Interest

The authors declare that the research was conducted in the absence of any commercial or financial relationships that could be construed as a potential conflict of interest.
